# Evaluating the effects of biocompatible cholinium ionic liquids on microbial lipid production by *Trichosporon fermentans*

**DOI:** 10.1186/s13068-015-0299-7

**Published:** 2015-08-15

**Authors:** Liping Liu, Yang Hu, Peng Wen, Ning Li, Minhua Zong, Beining Ou-Yang, Hong Wu

**Affiliations:** State Key Laboratory of Pulp and Paper Engineering, College of Light Industry and Food Sciences, South China University of Technology, 381 Wushan Rd., Tianhe District, Guangzhou, 510640 China; School of Biosciences and Bioengineering, Guangzhou Higher Education Mega Centre, South China University of Technology, 382 East Waihuan Rd., Panyu District, Guangzhou, 510640 China

**Keywords:** *Trichosporon fermentans*, Microbial lipid, Cholinium ionic liquid, Lipid production, Biofuel

## Abstract

**Background:**

Microbial lipid is a potential raw material for large-scale biodiesel production and lignocellulosic hydrolysate has been considered as promising low-cost substrate for lipid fermentation. Lignocellulosic biomass needs to be pretreated before enzymatic hydrolysis, and biocompatible cholinium ionic liquids (ILs) have been demonstrated to be highly efficient for pretreatment. However, the impact of these ILs residues in hydrolysates on downstream biotransformation remains unknown. Therefore, the influence of three typical cholinium ILs on the lipid production by *Trichosporon fermentans* was first investigated.

**Results:**

The cell growth of *T. fermentans* was stimulated in the presence of cholinium lysine ([Ch][Lys]) and cholinium serine ([Ch][Ser]), while the lipid accumulation was inhibited by [Ch][Lys]) and [Ch][Ser]. Both cell growth and lipid accumulation of *T. fermentans* were inhibited in the presence of cholinium acetate ([Ch][OAc]). Despite the reduction in lipid content, the lipid production by *T. fermentans* was improved in the presence of low concentrations of [Ch][Lys] (≤30 mM) and [Ch][Ser] (≤20 mM) due to the remarkable increase of biomass. It was found that cholinium cation had minor influence on lipid production. However, the anions of [Ch][Lys] and [Ch][Ser] could be assimilated as nitrogen source by *T. fermentans* and the reduced C/N ratio accounts for the inhibition of lipid accumulation, which could be alleviated by improving C/N ratio of medium. In addition, the anion of [Ch][OAc] could be metabolized by *T. fermentans*, leading to a rapid alkaline-pH shift and strong inhibition of lipid production. And this inhibitory effect on lipid production could be significantly reduced by controlling culture pH.

**Conclusions:**

The anions of [Ch][Lys], [Ch][Ser] and [Ch][OAc] play an important role in affecting the cell growth and lipid accumulation of *T. fermentans,* and the inhibition of these three ILs on lipid production can be alleviated by careful fermentation condition control. Hence, *T. fermentans* is a promising strain for microbial lipid production from cholinium ILs-pretreated lignocellulosic hydrolysates.

## Background

The rising cost of fossil fuels, coupled with concerns over the environmental impact of associated CO_2_ emission, calls for renewable and low-cost energy alternatives. Biodiesel has been proved to be an attractive alternative to the conventional fossil diesel [1]. However, most of the biodiesel was converted from edible oils, which has brought about the food vs. biofuel debate. Recently, because of the similar fatty acid composition to vegetable oils, microbial lipid has gained increasing interest in its use as feedstock for biodiesel production. Yet, high production cost of microbial lipid hinders its further application. To solve this problem, various low-cost substrates were adopted to lower its fermentation cost [2, 3]. Lignocellulosic biomass, the most abundant and renewable biomass resources in nature, is considered to be a promising low-cost raw material for biofuel production [4].

Before being effectively exploited, lignocellulosic biomass needs to be pretreated in order to enhance its accessibility to enzymatic hydrolysis [5]. Up to now, the most commonly used pretreatment method is dilute acid hydrolysis. Although this method can give considerable monosaccharide yield, special reactors are needed to resist the corrosion effect of acid on equipment. Moreover, the acid used will degrade sugars to inhibitors that are harmful for downstream microbial growth and product formation [6, 7]. Ionic liquids (ILs), a type of molten salts with melting points of <100°C, are composed of organic cations and organic or inorganic anions; and they are considered ‘ecofriendly’ because of negligible vapor pressure, non-flammability, high thermal, and chemical stability [8]. Since Rogers and co-workers [9] demonstrated the dissolving capacity of ILs to cellulose, ILs have emerged as promising solvents for lignocellulosic biomass pretreatment [10]. So far, imidazolium ILs have been proved to be the most effective ILs used in biomass pretreatment. Although this type of ILs can greatly increase the enzymatic hydrolysis rate of pretreated biomass, they are recently demonstrated to be harmful to microbes. For example, it was found that 1-butyl-3-methylimidazolium chloride ([Bmim]Cl) is about 300 times more toxic to *Vibrio fischeri* than acetone [11]. Additionally, when used for pretreatment of corn stover for bioethanol production, 1-ethyl-3-methylimidazolium acetate ([Emim][OAc]) at 52.4 mM could significantly inhibit *Saccharomyces cerevisiae*’s cell growth and ethanol production and there was a synergistic inhibitory effect between the anion and the cation [12]. Huang et al. further demonstrated that [Emim][OAc] could inhibit lipid production of oleaginous yeast *Rhodosporidium toruloides* due to the assimilation of acetate by the yeast which led to a rapid alkaline-pH shift [13]. Very lately, it was found that the presence of [Emim][OAc] could induce morphological changes of *S. cerevisiae*, which exhibited wrinkled, softened, and holed shapes [14]. Irrespective of their cytotoxicity, the non-biodegradable characteristics of imidazolium ILs would be another hamper to their wide application [15]. Therefore, it is of urgent need to find new solvents with more biocompatible and biodegradable properties for biomass pretreatment.

**Scheme 1:**
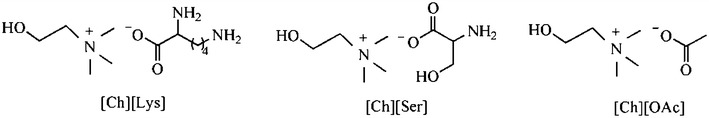
Chemical structures of the three cholinium ILs.

Recently, Hou et al. [16, 17] and Ninomiya et al. [18] reported a type of novel renewable cholinium ILs as highly effective solvents for lignocellulosic biomass pretreatment. For example, when [Ch][Lys] was used for pretreatment of rice straw at 90°C for 5 h, sugar yields of 84% for glucose and 42.1% for xylose were achieved. As biomass pretreatment solvents, it is inevitable that ILs will be left over at various concentrations in the lignocellulosic hydrolysates. To understand the effect of ILs on the downstream biotransformation will be helpful in assessing the possibility of using ILs-pretreated lignocellulosic hydrolysates for biofuel production. However, to date, there is no report about the impacts of cholinium ILs on microbial production of bio-based products. *Trichosporon fermentans* is an oleaginous yeast which can efficiently produce lipid in detoxified lignocellulosic hydrolysates [19, 20]. In this work, the effects of three typical cholinium ILs with robust lignocellulose pretreatment capability (i.e. cholinium lysine ([Ch][Lys]), cholinium serine ([Ch][Ser]), and cholinium acetate ([Ch][OAc], as shown in Scheme 1) on the cell growth and lipid accumulation of *T. fermentans* were firstly investigated. To give a deep insight into the influential mechanism, the sugar metabolism of cells and the effects of cation and anions of cholinium ILs on lipid production were further analyzed. This study will provide some valuable information for efficient application of cholinium ILs-pretreated lignocellulosic hydrolysates in biorefinery processes, particularly in microbial lipid production.

## Results and discussion

### Effects of cholinium ILs on cell growth and lipid accumulation of *T. fermentans*

A series of cholinium ILs have been synthesized and tested for lignocellulosic biomass pretreatment [17]. Most of these cholinium ILs, particularly [Ch][Lys], [Ch][Ser], and [Ch][OAc] had been demonstrated to be highly effective solvents for lignin dissolution which improved the accessibility of the remained polysaccharides to enzymatic hydrolysis [16, 18]. A previous work using [Emim][OAc] for lignocellulose pretreatment found that the concentrations of residual [Emim][OAc] remained in the subsequent enzymatic hydrolysates were up to 52 mM, depending on biomass regeneration process and washing conditions [12]. Accordingly, in this study, [Ch][Lys], [Ch][Ser], and [Ch][OAc] up to 60 mM were added into the fermentation media to investigate their effects on cell growth and lipid accumulation of *T. fermentans*.

**Fig. 1:**
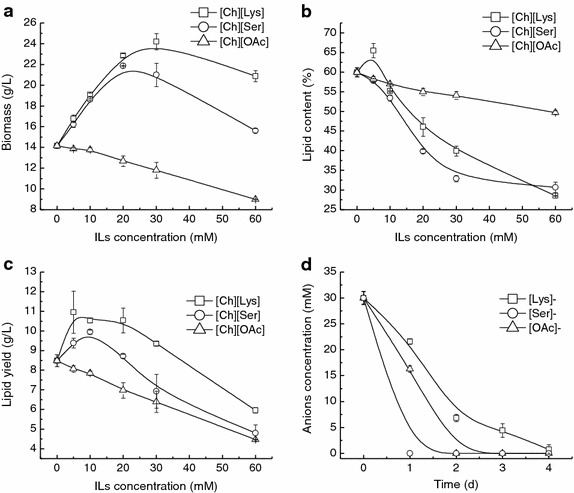
Lipid production by *T. fermentans* in the presence of [Ch][Lys], [Ch][Ser], and [Ch][OAc]. **a** Biomass, **b** lipid concent, and **c** lipid yield of *T. fermentans* in the presence of the cholinium ILs. **d** The evolution of the concentrations of [Lys]^−^, [Ser]^−^ and [OAc]^−^ in the fermentation media against time. The results are mean of two experiments, and *error bars* represent standard deviations from mean value.

As depicted in Fig. 1a, [Ch][Lys] could significantly stimulate the cell growth of *T. fermentans* except at 5 mM (*p* < 0.05). The biomass increased with the increase of [Ch][Lys] concentration and reached its maximum of 24.2 g/L at 30 mM, which was 171% of that obtained in the absence of IL (14.2 g/L). Further increase in the [Ch][Lys] concentration above 30 mM led to a slight drop in biomass. However, even at 60 mM, the biomass still reached 20.9 g/L which was 47.5% higher than the control. In contrast, the lipid content of *T. fermentans* decreased with the increase of [Ch][Lys] concentration (except at 5 mM), indicating that [Ch][Lys] had an inhibitory effect on lipid accumulation of *T. fermentans* (Fig. 1b). When [Ch][Lys] was at 60 mM, the lipid content of *T. fermentans* was only 28.5%, reduced by 52.4% compared with that obtained in the absence of the IL (28.5 vs. 59.9%). As can be seen in Fig. 1c, despite of the reduction in lipid content, the lipid production was still improved in the presence of low concentrations of [Ch][Lys] (≤30 mM), which was attributed to the remarkable increase of biomass. For [Ch][Ser], the impact of which on the cell growth and lipid accumulation of *T. fermentans* was quite similar to that of [Ch][Lys], with features of stimulating cell growth but inhibiting lipid accumulation, and the increase in biomass was significant except at 5 and 60 mM (*p* < 0.05), while the reduction in lipid content was significant except at 5 mM (*p* < 0.05). The lipid production was also improved in the presence of low concentrations of [Ch][Ser] (≤20 mM) due to the drastic increase of biomass. However, the biomass and lipid content of *T. fermentans* in the presence of various concentrations of [Ch][Ser] were all lower than those in the presence of equivalent amounts of [Ch][Lys]. Crépin et al. reported that lysine could be prematurely consumed by *S. cerevisiae* as nitrogen source [21]. It was also demonstrated that lysine could enhance the cell growth and ethanol production by regulating the nitrogen metabolism of *Saccharomyces pastorianus* [22]. Therefore, the stimulation effect of [Ch][Lys] and [Ch][Ser] on *T. fermentans*’ cell growth might be due to its assimilation of the anions of [Ch][Lys] and [Ch][Ser] for synthesis of cellular components, or improving the efficiency of nutrients uptake in the presence of additional ILs. Unlike [Ch][Lys] and [Ch][Ser], both cell growth and lipid accumulation of *T. fermentans* were inhibited by [Ch][OAc] and the biomass, lipid content, and lipid yield in the presence of 60 mM [Ch][OAc] were reduced by 36.5, 17.2, and 47.1%, respectively, compared with those of the control (Fig. 1a–c). Similar phenomenon was also observed in studying the effect of [Emim][OAc] on lipid production by *R. toruloides,* and the inhibition of [Emim][OAc] on cell growth and lipid accumulation was attributed to the assimilation of acetate which led to a rapid alkaline-pH shift [13].

**Fig. 2:**
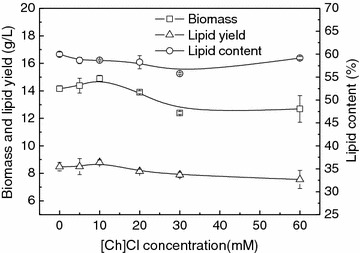
Effect of [Ch]Cl on the cell growth and lipid accumulation of *T. fermentans.* The results are mean of two experiments, and *error bars* represent standard deviations from mean value.

To test whether the anions of [Ch][Lys], [Ch][Ser], and [Ch][OAc] could be assimilated by *T. fermentans*, cells were cultured in the media containing 30 mM various ILs and 1 mL sample was taken daily to measure the anion concentration of the ILs. As can be seen in Fig. 1d, the concentrations of the three anions decreased with the increase of fermentation time and all the anions could be used up. The consumption rate of the anions followed the order: [Ser]^−^ > [OAc]^−^ > [Lys]^−^. It is known that both the cation and the anion of ILs can contribute to their toxicity [11, 12]. Hence, to understand the influence of cholinium cation on lipid production by *T. fermentans*, various concentrations of choline chloride ([Ch]Cl) were supplemented into the fermentation medium. As shown in Fig. 2, when [Ch]Cl was added at 10 mM, the biomass and lipid content were comparable to those of the control (14.9 vs. 14.2 g/L, 58.7 vs. 59.9%). Even in the presence of 60 mM [Ch]Cl, the biomass and lipid content still reached 12.7 g/L and 58.6%, respectively, indicating that cholinium cation has minor effect on cell growth and lipid accumulation of *T. fermentans*.

The effects of the selected cholinium ILs on the fatty acid composition of lipid produced by *T. fermentans* were also investigated, and the results were shown in Table 1. The major fatty acids of the lipid produced by *T. fermentans* in the absence of ILs were found to be oleic acid (C18:1), palmitic acid (C16:0), stearic acid (C18:0), and linoleic acid (C18:2), accounting for 60.6, 21.7, 11.3, and 5.3% of the total fatty acids. However, the presence of [Ch][Lys] significantly raised the relative content of C16:0 (*p* < 0.05). On the other hand, the relative content of C18:1 decreased with the increase of [Ch][Lys] and the reduction is significant when the IL concentration was ≥30 mM (*p* < 0.05). [Ch][Ser] could also improve the relative content of C16:0 while reduce the relative content of C18:1, but these alterations were not significant (*p* > 0.05) in most cases. The results suggest that these two ILs might inhibit the chain elongation and fatty acid desaturation in fatty acid synthesis. The impact of [Ch][OAc] on the fatty acid composition of lipid was not significant (*p* > 0.05), which was different from the result reported by Huang et al. that the relative content of C16:0 in the lipid produced by *R. toruloides* obviously increased but that of C18:1 decreased in the presence of [Emim][OAc] [13].

**Table 1:** Effect of the selected cholinium ILs on the fatty acid composition of lipid produced by *T. fermentans*

Culture conditions	Relative fatty acid content (%)				
	C 16:0	C 18:0	C 18:1	C 18:2	Others
Control					
0 mM	21.7 ± 1.9	11.3 ± 2.0	60.6 ± 0.4	5.3 ± 0.1	1.0 ± 0.1
[Ch][Lys]					
5 mM	28.9 ± 0.9	9.6 ± 0.5	55.3 ± 1.0	4.2 ± 0.5	1.9 ± 0.8
10 mM	32.1 ± 0.1	8.7 ± 0.1	54.9 ± 0.1	3.6 ± 0.1	0.7 ± 0.0
20 mM	32.0 ± 0.1	8.7 ± 0.4	52.9 ± 0.8	4.2 ± 0.2	2.1 ± 1.1
30 mM	34.5 ± 0.1	8.7 ± 0.2	51.1 ± 0.0	4.3 ± 0.3	1.5 ± 0.4
60 mM	34.4 ± 1.4	9.9 ± 2.4	47.5 ± 3.2	3.9 ± 0.0	1.5 ± 0.0
[Ch][Ser]					
5 mM	28.2 ± 2.1	9.1 ± 0.6	57.7 ± 3.0	5.0 ± 1.1	0.9 ± 0.4
10 mM	28.3 ± 0.2	8.5 ± 0.5	56.2 ± 1.6	5.5 ± 1.4	1.5 ± 0.9
20 mM	29.3 ± 0.1	7.5 ± 0.6	56.5 ± 0.1	4.8 ± 0.4	1.9 ± 0.4
30 mM	30.7 ± 0.7	8.1 ± 1.9	53.8 ± 3.4	5.9 ± 0.5	1.5 ± 0.3
60 mM	34.3 ± 0.7	7.4 ± 1.3	45.0 ± 3.8	6.5 ± 0.0	3.3 ± 2.6
[Ch][OAc]					
5 mM	24.4 ± 0.1	9.6 ± 0.2	60.3 ± 0.9	4.9 ± 0.3	0.7 ± 0.3
10 mM	24.4 ± 0.1	9.0 ± 0.0	60.9 ± 0.3	5.1 ± 0.1	1.0 ± 0.0
20 mM	25.4 ± 0.3	8.1 ± 0.0	60.3 ± 0.8	5.4 ± 0.3	0.9 ± 0.2
30 mM	23.2 ± 0.8	8.7 ± 0.2	59.6 ± 0.4	6.5 ± 0.3	1.9 ± 0.5
60 mM	25.0 ± 0.8	8.4 ± 0.3	59.0 ± 0.5	6.0 ± 0.1	1.6 ± 0.2

### Sugar consumption profile of *T. fermentans* in the presence of cholinium ILs

**Fig. 3:**
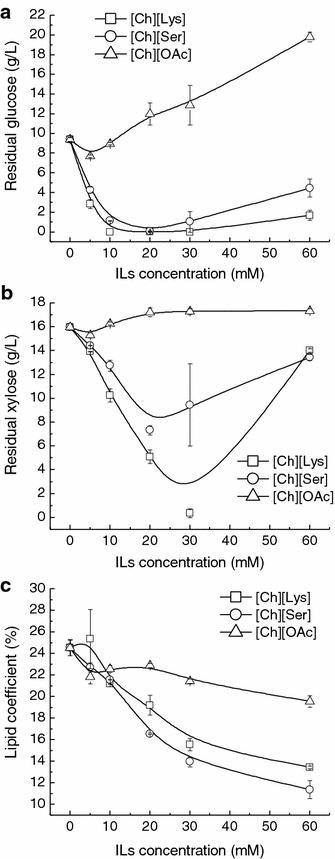
Effect of the selected cholinium ILs on sugar metabolism and lipid coefficient of *T. fermentans.* **a** Glucose consumption, **b** xylose consumption, and **c** lipid coefficient of *T. fermentans* in the presence of the cholinium ILs. The results are mean of two experiments, and *error bars* represent standard deviations from mean value.

To better understand the effect of the tested cholinium ILs on the cell growth and lipid accumulation of *T. fermentans*, the concentrations of residual sugars in the fermentation media after 4 days’ cultivation were measured. As shown in Fig. 3a, b, the residual glucose and xylose in the fermentation medium without ILs were 9.4 and 16.0 g/L, respectively. More glucose and xylose were consumed by *T. fermentans* in the presence of [Ch][Lys] and [Ch][Ser]. Specifically, the stimulation effect of [Ch][Lys] on sugar utilization was stronger than that of [Ch][Ser]. It was worth noting that when [Ch][Lys] was present at 30 mM, the glucose and xylose were almost exhausted by *T. fermentans*, which well explained the highest biomass achieved at this point. However, greater [Ch][Lys] in the media (above 30 mM) resulted in a slight drop in glucose consumption but a sharp decline in xylose consumption. Similar phenomenon was also observed in the presence of [Ch][Ser]. Interestingly, in the presence of [Ch][Lys] and [Ch][Ser], the extra sugar consumed by *T. fermentans* were not transformed into lipid indicated by the lower lipid coefficient in most cases compared with the control (Fig. 3c). In contrast, except that at its low concentration (≤10 mM), [Ch][OAc] showed inhibitory effect on both glucose and xylose metabolism of *T. fermentans*, and the inhibition increased with the increase of which concentration. Albeit more glucose and xylose were consumed when the concentration of [Ch][OAc] was below 10 mM, there was no improvement in the biomass and lipid content of *T. fermentans*, suggesting that the extra sugar consumed was not used for cell growth and lipid synthesis. Similar phenomenon was also observed in studying the influence of organic acids on *T. fermentans*’ lipid production [23]. It was reported that acetic acid (acetate) could interfere with yeast metabolism, which increased in the ATP requirement for cell maintenance [24, 25]. Hence, it is possible that the extra consumed sugars were used for synthesis of ATP. However, the actual mechanism still needs further investigation.

### The inhibitory mechanism of the selected cholinium ILs

#### Effect of C/N ratio on lipid production by *T. fermentans* in the presence of [Ch][Lys] and [Ch][Ser]

**Fig. 4:**
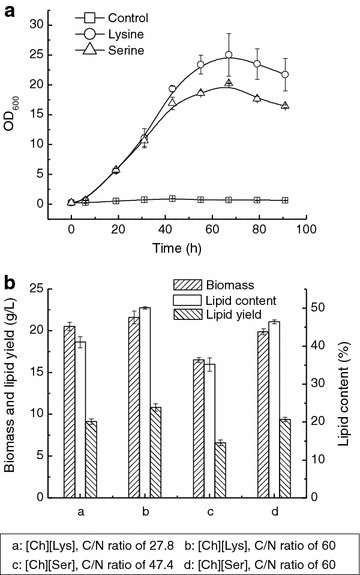
Effect of C/N ratio on the lipid production by *T. fermentans* in the presence of [Ch][Lys] or [Ch][Ser]. **a** Cell growth of *T. fermentans* in the medium containing 30 mM lysine or serine as sole nitrogen source. **b** Cell growth and lipid accumulation of *T. fermentans* in the media containing 30 mM [Ch][Lys] or [Ch][Ser] but with different C/N ratios. The results are mean of two experiments, and *error bars* represent standard deviations from mean value.

Previous reports showed that amino acids could be used as nitrogen source by yeasts [21, 22] and the above results demonstrated that the anions of [Ch][Lys] and [Ch][Ser] were assimilated by *T. fermentans*. To test whether these two anions can be utilized as nitrogen source by *T. fermentans*, cells were cultivated in the medium containing 30 mM lysine or serine as sole nitrogen source for 4 days and the OD_600_ values were recorded. As depicted in Fig. 4a, the OD_600_ values of the control (medium without any nitrogen source) remained almost unchanged during cultivation. In contrast, the OD_600_ values of culture broth supplied with 30 mM lysine or serine increased with time and reached its maximum of 25.04 or 20.26 at 67 h. After that, a slight decline in OD_600_ values was observed, indicating that lysine and serine were indeed used as nitrogen source by *T. fermentans*.

Generally, an excess of carbon substrate and a limiting amount of nitrogen in the medium are necessary for achieving high lipid accumulation in a microorganism [26]. It was found that 163 was the most suitable C/N ratio for lipid production by *T. fermentans* [27]. Undoubtedly, the existence of [Ch][Lys] or [Ch][Ser] would apparently reduce the C/N ratio of medium. For example, when 30 mM [Ch][Lys] or [Ch][Ser] was present, the C/N ratio of medium decreased from 163 to 27.8 and 47.4, respectively. Therefore, the inhibition of these two ILs on lipid accumulation of *T. fermentans* might be mainly due to the drastic reduction of C/N ratio. To testify this hypothesis, extra sugar was supplemented into the medium for elevating the C/N ratio. As indicated in Fig. 4b, a significant improvement in the lipid content (*p* < 0.05) at 30 mM [Ch][Lys] (50.1 vs. 41.1%) or [Ch][Ser] (46.4 vs. 35.2%) was observed when the C/N ratio was elevated to 60.

The results achieved here demonstrated that the stimulation effect of [Ch][Lys] and [Ch][Ser] on the cell growth of *T. fermentans* was attributed to the assimilation of amino acid anions of ILs as nitrogen source. Whereas, the inhibitory effect of these two ILs on the lipid accumulation of *T. fermentans* was due to the reduction of C/N ratio. And this inhibition could be efficiently relieved by regulating the C/N ratio of medium.

#### Effect of pH on lipid production by *T. fermentans* in the presence of [Ch][OAc]

**Fig. 5:**
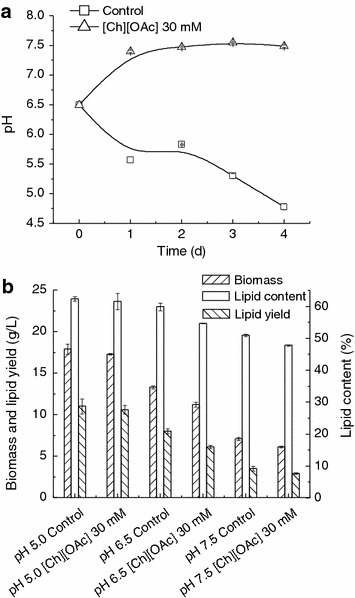
Effect of medium pH on the lipid production by *T. fermentans* in the presence of [Ch][OAc]. **a** pH profile of *T. fermentans* in the presence of [Ch][OAc]. **b** Cell growth and lipid accumulation of *T. fermentans* in the presence of [Ch][OAc] with pH control. The results are mean of two experiments, and *error bars* represent standard deviations from mean value.

It was reported that the inhibition effect of [Emim][OAc] on lipid production by *R. toruloides* was mainly due to a rapid alkaline-pH shift resulted from the assimilation of [OAc]^−^ [13]. In this study, the [OAc]^−^ of [Ch][OAc] was also metabolized by *T. fermentans*. Hence, the evolution of culture pH in the presence or absence of [Ch][OAc] was detected during fermentation. As can be seen in Fig. 5a, the culture pH shifted from 6.5 to 7.3 within 24 h and then tardily increased to 7.5 in the presence of 30 mM [Ch][OAc], while it dropped from 6.5 to 4.8 for the control. To investigate whether the pH change was the major reason for the inhibitory effect of [Ch][OAc] on lipid production by *T. fermentans*, cells were cultured under the controlled pH conditions in the presence of 30 mM [Ch][OAc]. Meanwhile, the control experiments were performed at the same conditions without IL. As shown in Fig. 5b, when cultures were maintained at pH 5.0, the values of biomass, lipid content, and lipid yield obtained in the presence of 30 mM [Ch][OAc] were the maximal, which were also very close to those achieved in the absence of [Ch][OAc]. For shake-flask fermentation of *T. fermentans* without pH control, the maximal values of biomass, lipid content, and lipid yield were found to be obtained at initial pH of 6.5 [27], which can be explained from the variation of culture pH as indicated in Fig. 5a (at approximately 5.0 during most of the time). The biomass, lipid content, and lipid yield decreased with the increase of controlled pH from 5.0 to 7.5, and the reduction in biomass and lipid yield was significant (*p* < 0.05) while it was insignificant for lipid content (*p* > 0.05). Therefore, the alkaline-pH change through assimilation of [OAc]^−^ accounts for the inhibitory effect of [Ch][OAc] on lipid production by *T. fermentans*.

Huang et al. previous demonstrated that *R. toruloides* is a robust lipid producer tolerating residual imidazolium ILs at low concentrations [13]. The results achieved here suggest that *T. fermentans* is a potential strain for microbial lipid production from cholinium ILs-pretreated lignocellulosic hydrolysates. Meanwhile, this work provides additional information for efficient application of cholinium ILs-pretreated lignocellulosic hydrolysates in biorefinery processes. It was reported that some yeasts tolerant to [Emim][OAc] were screened by using the media containing this IL [28], indicating that it is feasible to obtain IL-tolerant microorganism strains in nature. In addition, the tolerance of microorganisms to ILs could be also improved by domestication and/or genetic modification.

## Conclusions

Three cholinium ILs were investigated for their influences on lipid production by *T. fermentans.* Cholinium cation had minor influence on lipid production but the anions of [Ch][Lys], [Ch][Ser], and [Ch][OAc] were all assimilated by *T. fermentans.* The stimulation effect of [Ch][Lys] and [Ch][Ser] on cell growth was due to the use of anions as nitrogen source and the reduced C/N ratio accounted for the inhibition of these two ILs on lipid accumulation. The assimilation of [OAc]^−^ led to a rapid alkaline-pH shift and strong inhibition of lipid production. The inhibition of [Ch][Lys] and [Ch][Ser] on lipid accumulation can be alleviated by elevating the medium C/N ratio, while the suppression of [Ch][OAc] on cell growth and lipid accumulation can be eliminated greatly by culture pH control.

## Methods

### Strain and chemicals

Yeast strain *T. fermentans* CICC 1368 was obtained from the China Center of Industrial Collection and kept on wort agar at 4°C. [Ch][OAc], [Ch][Lys], and [Ch][Ser] were synthesized as described by Liu et al. [17] and [Ch]Cl was bought from Sinopharm Chemical Reagent Co., Ltd (Shanghai, China). Yeast extract (containing 4.0% ammonium-N and 10.0% total nitrogen) and peptone (containing 2.0% ammonium-N and 14.5% total nitrogen) were purchased from Huankai Biotech (Guangzhou, China). Lysine and serine were obtained from Yuanju Biotech (Shanghai, China). All other chemicals used were of analytical grade or chromatographically pure.

### Medium, precultivation, and cultivation

The precultivation medium contained glucose and xylose 20 g/L (ratio 2:1, wt/wt), yeast extract 10 g/L, and peptone 10 g/L. The composition of fermentation medium was as follows: glucose and xylose 60 g/L (ratio 2:1, wt/wt), peptone 1.05 g/L, yeast extract 0.375 g/L, KH_2_PO_4_ 2.0 g/L, MgSO_4_·7H_2_O 0.4 g/L, MnSO_4_·H_2_O 0.003 g/L, CuSO_4_·5H_2_O 0.0001 g/L. ILs were supplemented into the fermentation media at appropriate concentrations if necessary, and the final pH was adjusted to 6.5 with 4.0 M NaOH or 4.0 M HCl before sterilization. To investigate whether the anions of [Ch][Lys] and [Ch][Ser] could be used as sole nitrogen source by *T. fermentans*, 0.218 g lysine or 0.157 g serine instead of peptone and yeast extract was added into 50 mL fermentation medium, and the medium without any nitrogen source was used as the control. To test the effect of C/N ratio on lipid production by *T. fermentans*, the C/N ratio was improved from 27.8 or 47.4 to 60 through adding 6.52 or 3.82 g mixed sugars (glucose and xylose at ratio 2:1, wt/wt) into 50 mL fermentation medium in the presence of 30 mM [Ch][Lys] or [Ch][Ser]. To investigate the lipid production by *T. fermentans* in the presence of 30 mM [Ch][OAc] with pH control, fermentation medium was made with 20 mM phosphate buffer, pH 5.0, 6.5, and 7.5, instead of water. During fermentation process, pH was adjusted to specified value with 4.0 M NaOH or 4.0 M HCl when necessary.

Preculture was performed in a 250 mL conical flask containing 50 mL of precultivation medium at 28°C and 160 rpm for 24 h. Then, 5% seed culture (2.5 mL) was inoculated into a 250 mL conical flask containing 50 mL fermentation medium. To test whether the anions of [Ch][Lys] and [Ch][Lys] were used as nitrogen source by *T. fermentans*, cells in seed culture were collected by centrifugation and washed with sterile saline for three times before inoculation. Fermentation was carried out in a rotary shaker at 25°C and 160 rpm for 4 days. Experiments were done at least in duplicate and data were presented as mean ± standard error of mean of duplicate experiments.

### Analytical methods

The medium pH was detected by pH meter (Sartorius, Germany). Cells were harvested by centrifugation, washed twice with distilled water and dried at 105°C for 24 h to get a constant dry cell weight. Cellular lipid from dry biomass was extracted as described by Huang et al. [19]. Lipid yield was defined as the amount of lipid extracted from the cells in per liter fermentation broth (g/L). Lipid content was calculated as g lipid per g dry cell weight. Lipid coefficient was defined as g lipid produced per g sugar consumed and then multiplied by 100%. The fatty acid profile of the lipid was determined as described by Morrison and Smith [29]. The fatty acid methyl esters produced by saponifying followed by methylation of the lipid were analyzed by gas chromatography (GC-2010, Shimadzu Corporation, Japan) with flame-ionization detector and a DB-Wax capillary column (30 m × 0.25 mm × 0.25 µm, Agilent Technologies Inc., USA). The column temperature was maintained at 180°C for 2 min and then upgraded to 210°C at a rate of 5°C/min and kept for 11 min. Nitrogen was used as the carrier gas at 1.5 mL/min. Split ratio was 1:50 (v/v). The injector and the detector temperatures were set at 260 and 280°C, respectively.

Glucose, xylose, and acetate were measured by HPLC as described by Huang et al. [19]. [Lys]^−^ and [Ser]^−^ were analyzed by HPLC (Waters Corp., USA) using a photodiode array detector (Waters 996) and a Chirex 3126 (D)-penicil column (250 × 4.6 mm, Phenomenex Corp., CA, USA), and 1 mM CuSO_4_ aqueous solution was used as the mobile phase at 1.0 mL/min.

### Statistical analysis

All the experiments were performed at least in duplicate, and their average values with standard deviations were used for statistical analysis with SPSS 17.0 software for Windows (SPSS Statistics Inc., Chicago, IL, USA). One-way analysis of variance (ANOVA) and Tukey’s honestly significant differences (HSD) test were used to determine the significant differences of data at a 95% confidence interval.

## Abbreviations

IL: ionic liquid
[Ch][Lys]: cholinium lysine
[Ch][Ser]: cholinium serine
[Ch][OAc]: cholinium acetate
[Ch]Cl: choline chloride
C/N ratio: carbon to nitrogen molar ratio
